# Access of the LGBTQIA+ Population to Brazilian Public Primary Health Care Services: A Scoping Review

**DOI:** 10.1111/phn.70059

**Published:** 2026-01-12

**Authors:** Lariane Angel Cepas, Isadora Silva de Carvalho, Talita Morais Fernandes, Álvaro Francisco Lopes de Sousa, Carlos Arterio Sorgi, Eduardo Antonio Donadi, Ana Paula Morais Fernandes

**Affiliations:** ^1^ Department of General and Specialized Nursing Ribeirão Preto School of Nursing University of São Paulo Ribeirão Preto São Paulo Brazil; ^2^ Department of Medicine School of Medicine of Ribeirão Preto University of São Paulo Ribeirão Preto São Paulo Brazil; ^3^ Campus de Três Lagoas Universidade Federal de Mato Grosso do Sul Três Lagoas Mato Grosso do Sul Brazil; ^4^ Public Health Research Centre Comprehensive Health Research Center (CHRC), REAL NOVA University Lisbon Lisbon Portugal; ^5^ Department of Chemistry Ribeirão Preto Faculty of Philosophy Science, and Letters University of São Paulo Ribeirão Preto São Paulo Brazil

**Keywords:** access to primary healthcare, gender‐diverse, health inequities, healthcare disparities, public health, sexual and gender minorities

## Abstract

**Background:**

LGBTQIA+ individuals in Brazil face persistent inequities in accessing Primary Health Care (PHC), largely due to structural barriers, institutional discrimination, and limited professional training. Despite the existence of the National Policy for Integral LGBT Health, significant challenges remain in ensuring inclusive and equitable care.

**Objective:**

To map the available scientific evidence regarding the access of the LGBTQIA+ population to Brazilian public PHC services, identifying barriers, facilitators, and research gaps.

**Method:**

This scoping review followed the JBI framework and PRISMA‐ScR guidelines. Searches were conducted in MEDLINE/PubMed, Embase, Scopus, CINAHL, SciELO, LILACS for studies published between 2018 and 2023.

**Results:**

A total of 19 studies were included, predominantly qualitative (84.2%) and concentrated in the Northeast and Southeast regions, with no studies from the Central‐West. Most investigations prioritized healthcare professionals’ perspectives, particularly nurses and physicians, while the experiences of LGBTQIA+ users remained underrepresented. Barriers to access included institutional prejudice, lack of professional preparedness, and a narrow biomedical focus on HIV/aids. Facilitators involved social support networks, and the search for inclusive spaces. Research gaps persist, particularly regarding dissident gender identities.

**Conclusion:**

Strengthening inclusive policies, diversifying scientific production, and integrating sexual and gender diversity into professional training are essential to promoting equity and improving health outcomes.

## 1 Background

The formulation of public, social, and legislative policies requires a profound understanding of the relationships established within society, in order to ensure that such regulations meet the needs of diverse groups and promote social equity (Cardoso et al. 2024). In the field of health care, the LGBTQIAPN+ population (lesbians, gays, bisexuals, transgender and travestis, queer, intersex, asexual, pansexual, non‐binary, and other dissident identities) has historically faced marginalization, deprivation of rights, and structural violence, which compromise universal and equitable access to health services (Assis and Jesus 2012; Ferreira and Nascimento 2022).

Health inequities are avoidable disparities among individuals and social groups, resulting from structural and social injustices such as discrimination and minority exclusion (Whitehead and Dahlgren 2006; Guimarães et al. 2020). According to Braverman‐Bronstein et al. (2023), these inequities stem from multiple socioeconomic factors, including race, income, disability status, and non‐normative gender identity or sexual orientation. For the LGBTQIA+ population, such inequalities translate into greater vulnerability to mental disorders, substance abuse, and violence, in addition to difficulties in accessing health services due to stigmatizing care and the lack of professional preparedness to address the specific needs of this group (Guimarães et al. 2020; Pinto et al. 2021).

Scientific evidence demonstrates that the LGBTQIA+ population faces higher risks for chronic diseases and mental health problems, associated both with biological factors and with minority stress, which results from ongoing experiences of discrimination (Linhares et al. 2021; Chinazzo et al. 2021). The absence of comprehensive guidelines in health professional training contributes to the perpetuation of these inequalities, since curricula often neglect the needs of this population (da Silva Pereira et al. 2023).

The historical marginalization of LGBTQIA+ people in the health sector is linked to processes of pathologization, institutional discrimination, and shortcomings in the implementation of effective inclusive policies (Negreiros et al. 2019). Although progress has been made, such as the National Policy for Comprehensive LGBT Health (Brasil. Ministério da Saúde 2011), significant barriers remain in access to quality services and in the training of professionals for humanized care (da Silva Pereira et al. 2023). This exclusion undermines the principle of universality of the Unified Health System (SUS), as in practice, the care provided to the LGBTQIA+ population does not occur under equal conditions and is often marked by prejudice and unpreparedness (da Silva Pereira et al. 2023).

In addition to issues related to HIV/aids and the gender‐affirming process, which often dominate discussions about LGBTQIA+ health, it is essential to adopt a more comprehensive approach that considers the multiple factors influencing the well‐being of this population (da Silva Pereira et al. 2023; Cazeiro 2020). In Brazil, LGBTQIA+ individuals are among the social groups with the lowest access to health services, frequently facing discrimination within the very spaces of care (Pinto et al. 2021). This exclusion reinforces the need to strengthen Primary Health Care (PHC), the gateway to SUS, which plays a fundamental role in promoting health equity (Martins et al. 2019).

Access to PHC involves multiple dimensions, to define access, we will use the model proposed by Botelho and França Júnior (2018), which structures access to health services across different dimensions, including availability and supply (the existence of adequate and accessible services for this population), accessibility (geographical, economic, and structural barriers that affect access), accommodation (the capacity of services to meet the needs of this population), acceptability (the perceptions and experiences of LGBTQIA+ people regarding the care received), and effective use (the effectiveness of services in promoting the health of this population) (Botelho and França Júnior 2018). Theoretical models suggest that health care access should be analyzed as an interaction between supply‐side factors (availability, adequacy, and cost) and demand‐side factors (disease burden, attitudes, and knowledge about services) (Levesque et al. 2013; Camargo and Castanheira 2020). However, despite the importance of PHC in reducing inequalities, challenges persist regarding the LGBTQIA+ population's access to basic health services (Santana et al. 2020).

We also highlight the Minority Stress Theory (MST) as a key framework for this review. MST is a conceptual framework that examines how minoritized identities interact with structural and identity‐related stressors to shape health‐related experiences (Meyer 2003). According to MST, LGBTQIA+ people are exposed to additional stressors that extend beyond those encountered by non‐minority populations, which contribute to health inequities. These stressors include distal factors—such as prejudice, discrimination, and violence—and proximal factors, such as internalized stigma, fear of rejection, and identity concealment (Meyer 2003; Velasco et al. 2025). Within the Brazilian context, these processes are further influenced by structural determinants, including social inequalities, limited professional training, and non‐inclusive healthcare policies, which directly impact access to Primary Health Care (PHC). Although these stressors generate barriers to health equity, MST also highlights the role of coping strategies and social support in mitigating their negative effects. Thus, MST provides a valuable framework to analyze how general, distal, and proximal stressors intersect with individual and systemic factors, shaping the experiences of LGBTQIA+ people in accessing PHC services in Brazil.

Ensuring inclusive and effective care in PHC requires overcoming structural and interpersonal barriers that limit access for historically marginalized populations (Akotirene 2019; Hooks 2018). In Brazil, where profound social inequalities persist, knowledge remains limited regarding the challenges faced by this population within the Unified Health System (SUS) (Melo et al. 2020). In this context, it is essential to consolidate and synthesize the available scientific evidence to guide the formulation of public policies, the planning of actions, and the qualification of services aimed at the LGBTQIA+ population.

Against this background, a scoping review was conducted, as it represents a methodological approach particularly suitable for exploring emerging or underexplored areas, such as access of the LGBTQIA+ population to Primary Health Care in Brazil. This type of review allows mapping and synthesizing the evidence available, identifying knowledge gaps, and providing support for the development of evidence‐based public policies (Peters et al. 2024). The temporal scope between 2018 and 2023 is justified by the social and structural transformations intensified during this period, particularly due to the COVID‐19 pandemic and a challenging political climate that affected public health policies and the organization of health services. The pandemic context triggered significant challenges for health systems, exposing chronic vulnerabilities related to financing and management. These vulnerabilities directly impacted the organization of PHC and the access of historically marginalized populations, such as LGBTQIA+, making it urgent to understand how these effects were reflected in the scientific production.

Thus, this review not only highlights persistent gaps on the topic but also provides insights into the organization of actions at this level of care, supporting strategies that promote effective, qualified, and equitable healthcare. Accordingly, the present study aims to map the available scientific evidence regarding access of the LGBTQIA+ population to Primary Health Care in Brazil.

## 2 Methods

This study is a scoping review based on the principles outlined in the JBI Review Manual: (1) defining and aligning the review objectives and questions; (2) developing inclusion criteria aligned with the objectives and questions; (3) designing the search strategy; (4) conducting the evidence search; (5) selecting evidence; (6) extracting evidence; (7) analyzing evidence; (8) presenting results; and (9) synthesizing evidence in relation to the review objectives, drawing conclusions, and considering the implications of the findings (Peters et al. 2024).

In accordance with the JBI Manual for Evidence Synthesis, the protocol for this scoping review was developed in advance to ensure transparency. The protocol was registered on the Open Science Framework (OSF) in April 2024. The study report followed the Preferred Reporting Items for Systematic Reviews and Meta‐Analyses Extension for Scoping Reviews (PRISMA‐ScR) guidelines (Tricco et al. 2018).

The Population, Concept, and Context (PCC) framework guided the definition of the review objectives, research questions, and search strategy, with P (Population) = LGBTQIA+ population, C (Concept) = access to health services, and C (Context) = Primary Health Care in Brazil. This framework supported the formulation of the central research question: “How has access to Primary Health Care by the LGBTQIA+ population in Brazil been described?” Secondary questions were developed to deepen the analysis of the findings and structure data extraction, allowing a more comprehensive understanding of different perspectives on the phenomenon: “What are the perceptions of the LGBTQIA+ population regarding their access to Primary Health Care in Brazil?”; “What are health professionals’ perceptions of care provided to the LGBTQIA+ population in Primary Health Care in Brazil?”; and “Which barriers and facilitators have been identified in the Brazilian health system concerning access of the LGBTQIA+ population to Primary Health Care?” These questions guided searches for articles indexed in the MEDLINE/PubMed, Embase, Scopus, CINAHL, SciELO, and LILACS databases.

The search strategy was structured based on the PCC framework and developed using Medical Subject Headings (MeSH) and Health Sciences Descriptors (DeCS), in addition to keywords, combined with the Boolean operators “AND” and “OR.” The strategy was adapted for each database, considering language and system‐specific features, and reviewed by a specialist librarian.

Inclusion criteria were: primary studies, without language restriction, published between 2018 and 2023, and indexed in the aforementioned databases. Exclusion criteria included protocols, systematic reviews, studies whose titles and abstracts did not address the research question, studies not including perspectives of LGBTQIA+ individuals or health professionals providing care to this population, studies conducted outside public Primary Health Care services in Brazil, studies not available in full text, and duplicate studies across databases. Experience reports, opinion articles, editorials, gray literature, and other publications without empirical data were also excluded. Although community reports, theses, technical documents, and other forms of gray literature can capture innovative and community‐led initiatives, particularly in LGBTQIA+ health, we chose to limit inclusion to peer‐reviewed primary studies indexed in scientific databases. This decision sought to ensure a minimum level of methodological rigor, transparency, and retrievability across sources, facilitating a more robust comparison of study designs and findings.

Database searches were conducted in April 2024 and documented with details regarding the database, search date, strategy (combination of descriptors and/or keywords), limits, and number of articles retrieved (Appendix 1).

**FIGURE 1:**
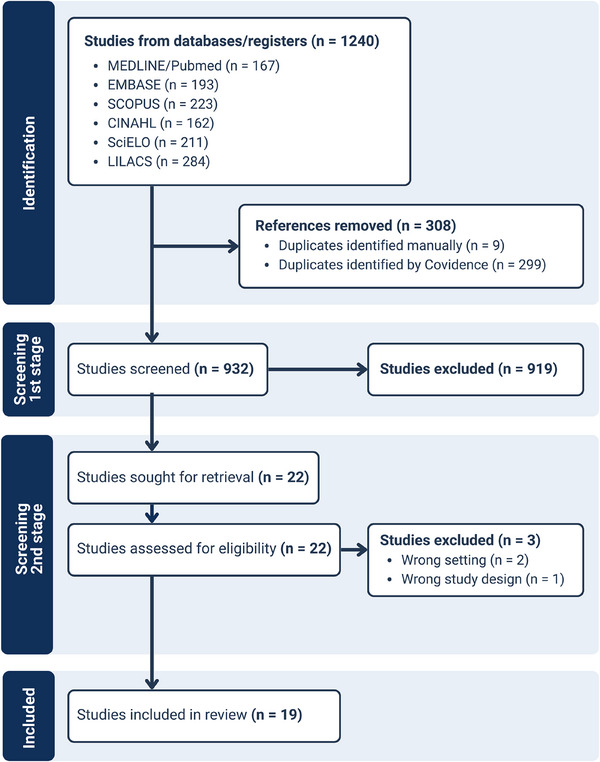
Flowchart illustrating the study selection and extraction process, developed based on the PRISMA‐ScR flow (Tricco et al. 2018). [Colour figure can be viewed at wileyonlinelibrary.com]

References were exported to Covidence for the review process, which consisted of two screening stages. The first involved screening titles and abstracts to assess eligibility, and the second involved full‐text reading of studies deemed eligible in the first stage. Both stages were conducted by two independent reviewers, and discrepancies were resolved through discussion or with the participation of an additional reviewer until consensus was reached, followed by final study selection. The study report adhered to the PRISMA‐ScR guidelines (Tricco et al. 2018). The flow diagram of this process is presented in Figure 1.

Key information from each selected article was extracted using a data extraction form specifically developed for this review based on the JBI model (Peters et al. 2024), to guide descriptive and narrative/critical analyses of the included studies. Data extraction was performed by one reviewer and verified by a second reviewer to ensure accuracy and consistency.

The data extraction form included the following information: title, author, year of publication, study design, objectives, study population composing the samples, Primary Health Care services, state or region where the study was conducted, and main findings.

After data extraction, results were categorized and discussed by the authors. The primary extraction category focused on barriers and facilitators to access, identified from the perspectives of LGBTQIA+ individuals, health professionals, healthcare services, and the health system. Findings are presented in tables and figures, as well as in narrative format, addressing bibliometric aspects and answering the research questions that guided this review.

Methodological quality of included studies was not assessed, as this review considered only articles indexed in scientific databases, ensuring a minimum level of methodological rigor. This approach aligns with the objectives of a scoping review, which aim to map the extent and nature of available evidence rather than to critically appraise study quality.

## 3 Results

A total of 1244 articles were identified across the databases. Of these, 308 were excluded as duplicates (299 detected automatically by Covidence and 9 identified manually). Following title and abstract screening, 919 articles were excluded, and an additional three were excluded after full‐text review. After this process, 19 articles were selected for inclusion in the study (Figure 1).

Below, in Table 1, the 19 studies considered eligible to compose the sample are presented, regarding the authors, year of publication, geographic location of the study, objective, method, design, and participant profile.

**TABLE 1:** Characteristics of the studies comprising the scoping review sample, in terms of authors, year of publication, geographic location of the study, objective, method, design, and participant profile.

Authors/year/setting	Objective	Method/design	Participants
de Oliveira Ferreira et al. (2018), Teresina‐PI	To understand the dimensions of access and comprehensive care in the Unified Health System from the gender diversity perspective	Qualitative/Exploratory study	19 sexual and gender minority individuals: lesbians (4), gay men (4), transvestites (6), and transgender women (5)
Oliveira et al. (2018), Cajazeiras‐PB	To analyze the access of Lesbian, Gay, Bisexual, and Transvestite/Transsexual to the Basic Units of Family Health from the perspective of professionals of the Family Health Team	Quantitative/Exploratory descriptive study	54 health professionals part of a Family Health Team (physicians, nurses, dentists, nursing technicians, oral health technicians, and community health agents)
Fernandes et al. (2019), Vitória da Conquista‐BH	To evaluate access to cytological examination of uterine cancer in the Family Health Strategy (ESF), in municipalities in a health region in Bahia	Qualitative/Exploratory study	70 community health agents and nurses
Paulino et al. (2019), Uberlândia and Belo Horizonte‐MG	To identify discourses on access and quality of comprehensive health care for LGBT population among doctors of the Family Health Strategy	Qualitative/Exploratory descriptive study	15 physicians from the Family Health Strategy
Silva et al. (2019), Florianópolis‐SC	To understand the social representations of Primary Health Care workers about lesbians, gays, bisexuals, transvestites, and transsexuals	Qualitative/Exploratory descriptive study	15 health professionals: Nursing assistants or technicians and/or oral health technicians (4), dentists (3), nurses (3), physicians (2), community health agents (2), and administrative assistant (1)
Araújo et al. (2020), unspecified city in the Northeast region	To analyze the meanings attributed by nurses in primary care about the knowledge and practice of welcoming the LGBT population	Qualitative/Exploratory descriptive study	20 nurses
Ferreira and Bonan (2021), Teresina‐PI	To analyze reports of professionals who assist the LGBTT population in the Family Health Strategy	Qualitative/Descriptive study	32 health professionals: physicians, nurses, dentists, dental health technicians and assistants, nursing technicians and assistants, community health agents, coordinator, psychologist, physiotherapist, and nutritionist
Reis et al. (2021), Manaus‐AM	To understand the nurses‐created meanings regarding the user embracement of transvestite and transsexual people in primary care	Qualitative/Exploratory descriptive study	4 nurses
Torres et al. (2021), National	To characterize the LGBT+ population during the COVID‐19 pandemic and to specify the characteristics of the COVID‐19 pandemic in this population	Quantitative/Descriptive cross‐sectional study	976 LGBT+ individuals
Costa‐Val et al. (2022), Ouro Preto‐MG	To comprehend the aspects related to the access and care of the LGBT population from the perspective of professionals from Basic Health Units	Qualitative/Exploratory study	15 health professionals: physicians (4); community health agents (5); technical nurses (2); nurses (2); administrative agents (2)
Gomes et al. (2022), Rio de Janeiro‐RJ	To identify and discuss the reasons that restrict or hinder the accessibility of transsexuals to primary health services	Qualitative/Exploratory descriptive study	12 transsexuals
Ketzer et al. (2022), Porto Alegre‐RS	To analyze the narratives of lesbian women about sexual and reproductive health care in Primary Health Care services	Qualitative/Exploratory descriptive study	10 lesbian women
Miskolci et al. (2022), National (São Paulo‐SP, Curitiba‐PR, Florianópolis‐SC, Porto Alegre‐RS)	To analyze the current challenges to the health of LGBTI+ individuals and other sexual and gender minorities in the Brazilian context	Qualitative/Mixed‐methods triangulation	134 participants: managers, health professionals, and LGBTI+ individuals
Miskolci and Pereira (2022), São Paulo‐SP	To identify and analyze how health professionals working in primary care in the city of São Paulo perceive access to health care for LGBTI+ individuals	Qualitative/Digital ethnography	29 health professionals: administrators, doctors, nurses, and nursing technicians/assistants
Crenitte et al. (2023), National	To compare the variables of access to healthcare between the LGBT+ population aged 50 and over and those non‐LGBT+	Quantitative/descriptive cross‐sectional study	6693 participants: LGBT+ (1332) and non‐LGBT+ (5361)
Gomes et al. (2023), National	To know the experiences and demands of transgender people when seeking primary health care services	Qualitative/Descriptive study	20 transgender individuals: transgender men (10), transgender women (10)
Lopes et al. (2023), Rio de Janeiro, RJ	To evaluate the access to reception for the LGBTQIA+ population by health professionals from a Municipal Health Center in the city of Rio de Janeiro	Qualitative/Descriptive study	72 professionals from the Family Health Strategy: physicians (8), nurses (8), nursing technicians (16), community health workers (37), dentists (3)
Mendes et al. (2023), Fortaleza, CE	To investigate the actions of nurses in regard to lesbian and bisexual women in the context of the National Policy for the Integral Health of Lesbians, Gays, Bisexuals, Cross‐Dressers, and Transgender Persons	Qualitative/Descriptive study	25 nurses from the Family Health Strategy
Paiva et al. (2023), São Paulo (state)	To analyze the role of Family Health Strategy (FHS) nurses in the health care of LGBT+ individuals	Qualitative/Descriptive study	14 nurses from the Family Health Strategy

*Note*: Regarding the methodological design, 16 (84.2%) studies were categorized as qualitative, and 3 (15.8%) as quantitative.

**FIGURE 2:**
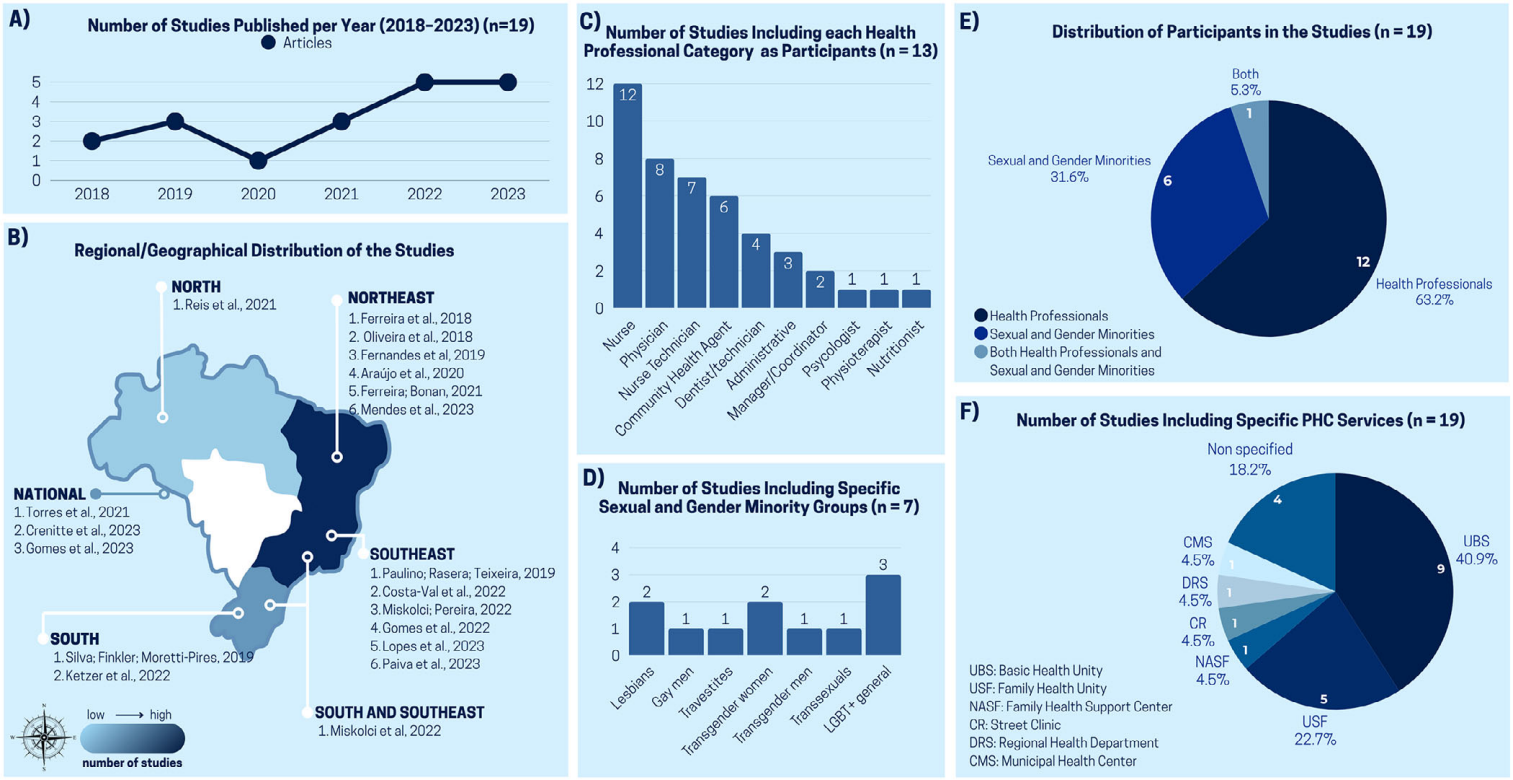
Synoptic overview of the studies included in the scoping review, regarding year of publication, geopolitical distribution, participants, study setting, and results. [Colour figure can be viewed at wileyonlinelibrary.com]

Between 2018 and 2023, 19 studies related to the topic of this review were published in the selected databases. A growth trend can be observed over this period. In 2018, two articles were published, increasing to three in 2019. In 2020, there was a decrease to only one article. From 2021 onward, a progressive increase was observed: three articles in 2021, followed by five publications in 2022, a number that remained constant in 2023 (Figure 2A).

Regarding the geopolitical distribution (Figure 2B), most studies were conducted in the Northeast (31.6%) and Southeast (31.6%) regions, followed by the South (10.5%) and North (5.3%). Three national studies were identified (15.7%), and one study was conducted across both the South and Southeast regions (5.3%). It should be noted that, based on the search strategy used in this study and the application of eligibility criteria, no publications were found from the Central‐West region.

Most of the topics addressed in the studies included access to PHC, barriers to access, reception and care practices in PHC, sexual and reproductive health, and social and political representations of access and/or care in PHC from the perspective of healthcare professionals, the LGBTQIA+ population, or both.

Regarding study participants: 12 studies (63.2%) included healthcare professionals, six studies (31.5%) included LGBTQIA+ individuals, and only one study (5.3%) included both healthcare professionals and the LGBTQIA+ population (Figure 2E).

Among the studies that included the LGBTQIA+ population, two included only transgender individuals, one included only lesbian women, one included lesbian women, gay men, travestis, and trans women, and two included the entire LGBTQIA+ population without distinguishing specific groups (Figure 2D).

Concerning the frequency of participation of different healthcare professional categories in the analyzed studies (*n* = 13), nurses were the most frequently included, appearing in 12 studies. They were followed by physicians (8), nursing technicians (7), and community health agents (6). Other categories appeared less frequently: dentists/dental health technicians (4); administrative professionals (3); managers or coordinators (2); and psychologists, physiotherapists, and nutritionists, each mentioned once (Figure 2C).

Among the 19 studies analyzed, most included specific PHC services. The Basic Health Unit (UBS) was the most frequently mentioned, representing 40.9% of inclusions (nine studies). Next were Family Health Units (USF), with 22.7% (five studies). Other service modalities were mentioned once each (4.5%): Family Health Support Centers (NASF), Street Clinics (CR), Regional Health Departments (DRS), and Municipal Health Centers (CMS). In four studies (18.2%), the PHC service was not specified, or they analyzed access to PHC without focusing on a specific service as the study setting (Figure 2F).

**FIGURE 3:**
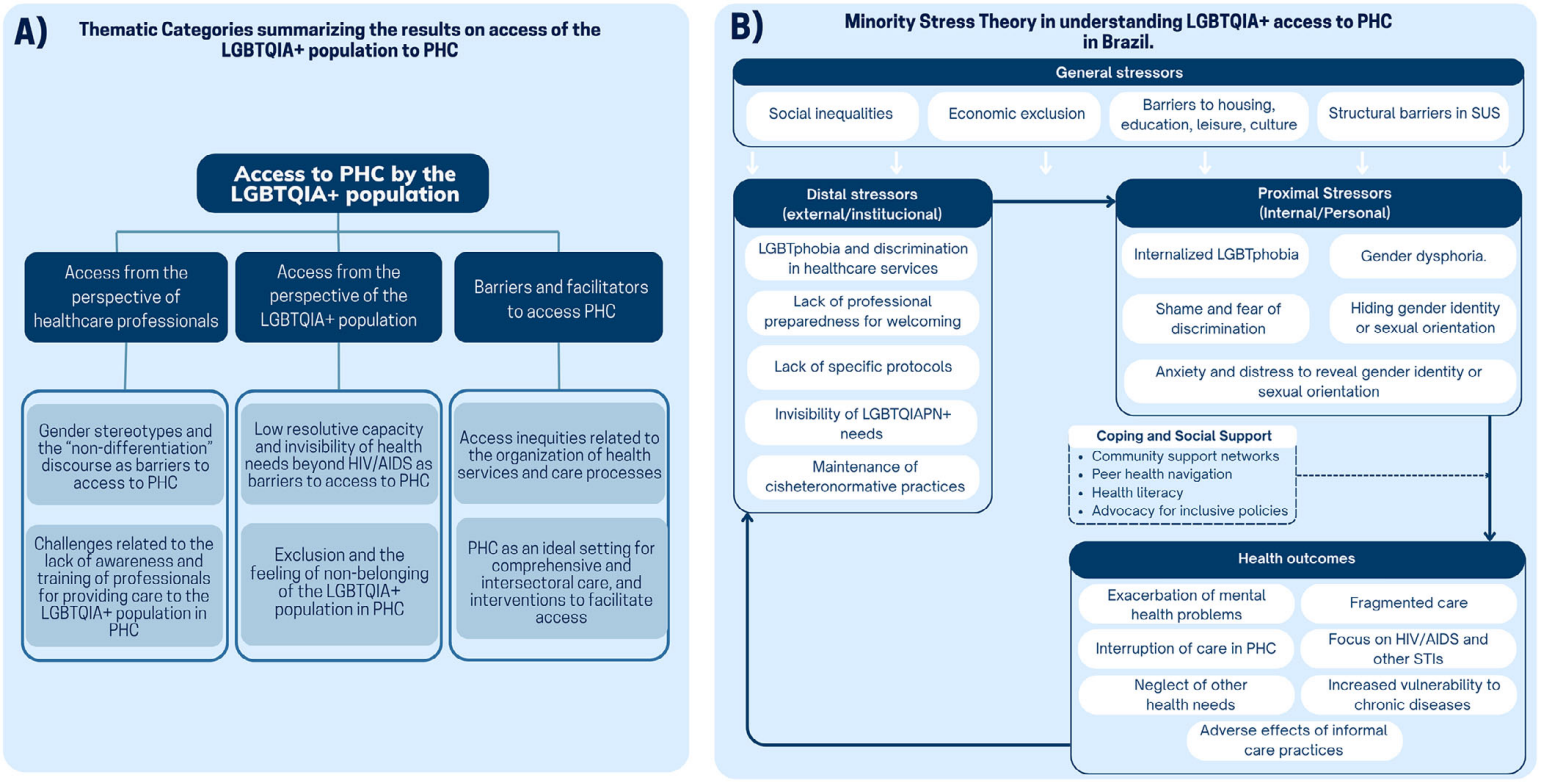
Synoptic overview of the results on access to PHC. [Colour figure can be viewed at wileyonlinelibrary.com]

Figure 3A presents the identified thematic categories along with their respective subcategories, which emerged from an in‐depth analysis of the included studies. The three main categories were organized to address the research questions of the review: (1) Access from the perspective of healthcare professionals, (2) Access from the perspective of the LGBTQIA+ population, and (3) Challenges and solutions for access to PHC.

In the 19 studies analyzed on access to PHC for the LGBTQIA+ population, three central thematic categories emerge, reflecting both the perspectives of professionals and service users, as well as structural challenges and potential solutions to improve access and quality of care (Figure 3A). These findings can be further understood through the application of the MST, which differentiates general, distal, and proximal stressors and illustrates how these factors interact to influence health outcomes, as well as the coping and support strategies adopted by the population (Figure 3B).

## 4 Discussion

The findings of this scoping review highlight significant gaps and inequalities in the scientific production regarding access to PHC for the LGBTQIA+ population in Brazil. The predominance of healthcare professionals over LGBTQIA+ individuals as study participants underscores the research focus on biomedical and institutional perspectives, often overlooking the lived experiences and agency of marginalized populations. This asymmetry contributes to the perpetuation of invisibility and silencing of these groups in knowledge production and public policy formulation spaces (Santos et al. 2024). The fact that only one study simultaneously included both healthcare professionals and LGBTQIA+ individuals reveals the persistent fragmentation between those who provide care and those who experience it, hindering a broader and more intersectional understanding of challenges in accessing PHC.

Although studies involving the LGBTQIA+ population analyze different segments, there is a predominant concentration on socially more visible groups, such as lesbian women, gay men, and transgender individuals. In contrast, identities such as non‐binary, bisexual, intersex, and other dissident expressions of gender and sexuality remain underrepresented or invisibilized in the scientific literature. This limitation underscores the urgent need to broaden research scopes to encompass the diversity of experiences and needs within the LGBTQIA+ community itself (Carvalho and Barreto 2021).

The higher frequency of participation of nurses and physicians—professionals traditionally linked to Basic Health Units, which represent the conventional model of primary healthcare in Brazil—highlights the predominance of traditional PHC approaches in scientific production. In contrast, the Family Health Strategy (ESF) and Family Health Support Centers (NASF) correspond to newer, still nationally expanding models of care, designed to provide more comprehensive, multidisciplinary, and community‐oriented services. However, research involving professionals from these newer arrangements, including multiprofessional teams (eMulti), remains scarce. The limited inclusion of specific services, such as Street Clinics and NASF, further underscores the need to expand the research focus toward care models that are more responsive to the specificities and vulnerabilities of the LGBTQIA+ population. The persistent focus on traditional primary healthcare modalities, such as Basic Health Units, suggests challenges in incorporating more inclusive, interdisciplinary, and welcoming perspectives—approaches that are essential for implementing public health policies that ensure universal and equitable access, as mandated by the Brazilian Unified Health System (SUS) (Campos et al. 2020; Bispo Júnior and Almeida 2023).

Moreover, the geographic distribution of scientific production revealed a marked concentration in the Southeast and Northeast regions, with a complete absence of studies from the Central‐West region and minimal representation from the North. This panorama reflects historical structural inequalities, combining lower research investment, scarcity of graduate programs, and weaknesses in funding mechanisms provided by research support agencies in these regions (Sidone et al. 2016). Added to this is the political and territorial complexity of these regions, characterized by borders, intense migration, and the presence of indigenous and traditional populations, who remain systematically invisibilized and insufficiently addressed in public policies and LGBTQIA+ health research (Sidone et al. 2016).

### 4.1 Access From the Perspective of the LGBTQIA± Population

From the perspective of studies involving LGBTQIA+ users, PHC continues to exhibit shortcomings in providing comprehensive and responsive care to the multiple needs of this population (de Oliveira Ferreira et al. 2018; Torres et al. 2021; Gomes et al. 2022; Ketzer et al. 2022; Miskolci et al. 2022; Crenitte et al. 2023; Gomes et al. 2023). The prevailing model remains centered on the prevention of HIV/aids and other sexually transmitted infections (STIs), while other important health needs—such as mental health, gynecological care for lesbian and bisexual women, and gender‐affirming care for transgender and intersex individuals—remain largely neglected (Gomes et al. 2022; Ketzer et al. 2022; Miskolci et al. 2022).

This phenomenon is reinforced by symbolic exclusion and the production of a sense of non‐belonging, which deters LGBTQIA+ individuals from engaging with healthcare services, whether through explicit discriminatory practices or professional unpreparedness resulting in inadequate and even harmful reception (Gomes et al. 2022; de Oliveira Ferreira et al. 2018). These barriers correspond to what Levesque et al. (2013) describe as a “lack of acceptability,” wherein services are not perceived as responsive or respectful toward the specific needs of users.

Furthermore, care is often isolated within specialized services, such as Testing and Counseling Centers (CTAs) and transition clinics, a phenomenon that reinforces the disengagement of LGBTQIA+ individuals from PHC and undermines its function as the first point of contact and entry into the health system. Such isolation compromises continuity of care, the addressing of other health needs, and the preventive and health promotion activities characteristic of PHC as a privileged space for comprehensive and longitudinal care. The invisibility of needs and the institutional violence experienced corroborate evidence on the deleterious effects of discrimination on mental health and care‐seeking behaviors (Chinazzo et al. 2021).

From the perspective of Minority Stress Theory, symbolic exclusion and institutional discrimination faced by the LGBTQIA+ population constitute distal stressors—that is, external, concrete factors of prejudice and marginalization within healthcare settings (Chinazzo et al. 2021; Miskolci et al. 2022). These external manifestations generate internal consequences—proximal stressors—such as feelings of non‐belonging, fear, anxiety, and internalized homonegativity (Ferreira and Bonan 2021; Oliveira et al. 2018).

The sense of non‐belonging arises from the perception that services are neither welcoming nor responsive to the specific needs of LGBTQIA+ individuals, reinforcing voluntary disengagement and avoidance of care (Gomes et al. 2022; Ketzer et al. 2022). Associated proximal stressors intensify negative impacts on mental health and well‐being, creating a vicious cycle of exclusion and vulnerability (Levesque et al. 2013).

Therefore, exclusion as a distal stressor does not operate in isolation but acts as a trigger for internal suffering that limits care‐seeking behaviors and compromises comprehensive access to healthcare. These findings underscore the urgent need for interventions that foster welcoming, inclusive, and contextually aware environments, capable of breaking the cycle of exclusion and stress (Araújo et al. 2020; Reis et al. 2021).

### 4.2 Access From the Perspective of Healthcare Professionals

Studies examining healthcare professionals’ perspectives on providing care to the LGBTQIA+ population in Primary Health Care (PHC) reveal that such care is profoundly influenced by gender stereotypes, personal beliefs, and the discourse of “non‐differentiation” (Oliveira et al. 2018; Fernandes et al. 2019; Paulino et al. 2019; Silva et al. 2019; Araújo et al. 2020; Ferreira and Bonan 2021; Reis et al. 2021; Costa‐Val et al. 2022; Miskolci et al. 2022; Miskolci and Pereira 2022; Lopes et al. 2023; Mendes et al. 2023; Paiva et al. 2023). The latter stance, which appears neutral, tends to render invisible the specificities and vulnerabilities of this population, contributing to their marginalization within healthcare services (Paulino et al. 2019; Costa‐Val et al. 2022).

From the perspective of Minority Stress Theory, these factors constitute distal stressors—external to the individual—that manifest as institutional prejudice and inadequate reception, creating an adverse environment for LGBTQIA+ individuals. Insufficient training and awareness among healthcare professionals perpetuate clinical practices grounded in heteronormativity and cisnormativity, exacerbating stigma and contributing to the maintenance of chronic stress experienced by these populations (Ferreira and Bonan 2021; Miskolci et al. 2022). The discourse of “not knowing,” frequently employed to justify inadequate care, reflects resistance to change and gaps in professional updating, which could otherwise mitigate suffering and improve quality of care (Paulino et al. 2019).

Furthermore, these external factors give rise to proximal stressors—internal to the individual—such as anxiety, internalized homonegativity, and feelings of vulnerability when seeking services perceived as unwelcoming. This dynamic reinforces cycles of exclusion and has detrimental effects on the mental and physical health of LGBTQIA+ individuals (Oliveira et al. 2018; Araújo et al. 2020; Reis et al. 2021).

Finally, these findings align with critiques of traditional biomedical education, which has historically privileged models centered on the biological body and gender binarism, without integrating the social dimensions that shape LGBTQIA+ health (Ayres 2004; Silva and Almeida 2024). This underscores the urgent need to incorporate content on sexual and gender diversity into healthcare education and continuing professional development, which is essential for reducing distal stressors, fostering more inclusive environments, and mitigating proximal stressors in access to care.

### 4.3 Barriers and Facilitators to Access

Studies on the access of LGBTQIA+ populations to PHC reveal significant structural challenges related to the organization of services and care processes, such as information systems that fail to account for diverse gender identities, the absence of specific protocols, and the lack of effective public policies (Gomes et al. 2022; Miskolci et al. 2022). Nonetheless, these studies also highlight PHC as a strategic and promising space for the promotion of comprehensive, interdisciplinary, and equity‐oriented care (Costa‐Val et al. 2022; Miskolci and Pereira 2022). To realize this potential, it is imperative to implement interventions that go beyond technical training, encompassing the ethical, cultural, and political sensitization of healthcare professionals for the care of LGBTQIA+ populations (Oliveira et al. 2018).

The LGBTQIA+ population develops various coping strategies to deal with challenges encountered in PHC. A prominent strategy is the active search for welcoming spaces and healthcare professionals sensitive to their gender identities and sexual orientations, which enhances trust in the care provided (Araújo et al. 2020; Costa‐Val et al. 2022). Moreover, the formation of social and community networks plays an important role in strengthening mental health and overcoming stigma, by promoting emotional support and the exchange of experiences (Miskolci and Pereira 2022; Ferreira and Bonan 2021). The population also employs strategies of self‐defense and resistance, whether through critical dialogue with healthcare professionals in the face of discriminatory practices or through engagement in social movements that promote rights and visibility (Gomes et al. 2022; Reis et al. 2021). Another relevant aspect is the use of informal channels and non‐official groups to obtain information and support, particularly in the absence of adequate formal guidance (Miskolci et al. 2022; de Oliveira Ferreira et al. 2018).

To improve access and quality of care in PHC, it is essential to create or adapt specific protocols and information systems that incorporate diverse gender identities and sexual orientations, thereby fostering individualized and respectful care (Gomes et al. 2022; Miskolci et al. 2022). Strengthening intersectoral networks among the fields of health, social assistance, education, and human rights contributes to ensuring comprehensive and multidimensional care (Miskolci and Pereira 2022; Lopes et al. 2023). In addition, the promotion of inclusive and plural welcoming spaces aligned with the concept of expanded care, which considers not only the biomedical dimension but also the social determinants of health, subjectivities, and life trajectories of individuals, is crucial to achieving equity in healthcare provision (Araújo et al. 2020; Ketzer et al. 2022; Costa‐Val et al. 2022; Ayres 2004). Finally, the development of specific public policies, supported by effective funding and monitoring, is indispensable to expand access and improve the quality of PHC for LGBTQIA+ populations (Miskolci et al. 2022; Gomes et al. 2022; Torres et al. 2021).

### 4.4 MST in Understanding LGBTQIA± Access to PHC in Brazil

Figure 3B illustrates the application of MST in understanding LGBTQIA+ access to PHC in Brazil. The model differentiates general, distal, and proximal stressors, demonstrating how these factors interact to influence health outcomes and the coping and support strategies adopted by the population.

At the first level, general stressors encompass broad social and structural factors that, while affecting the population at large, disproportionately impact LGBTQIA+ individuals. These include persistent socioeconomic inequalities, exclusion across multiple domains such as housing, education, leisure, and cultural access, as well as institutional limitations within the Brazilian Unified Health System (SUS) (Miskolci et al. 2022; de Oliveira Ferreira et al. 2018; Araújo et al. 2020). This structural context creates a foundation of vulnerability and hinders full access to and utilization of PHC services for this population (Miskolci and Pereira 2022; Oliveira et al. 2018).

At the second level, distal stressors correspond to experiences occurring within institutional and social contexts, representing significant barriers to healthcare. These include explicit discriminatory practices, institutionalized LGBTQIA+ phobia, the perpetuation of cisheteronormative models that marginalize diverse identities, lack of welcoming environments, healthcare professionals’ unpreparedness to adequately serve this population, and the absence of LGBTQIA+‐specific clinical protocols (Gomes et al. 2022; Reis et al. 2021; Lopes et al. 2023; Fernandes et al. 2019). These factors generate fear, stigma, and social exclusion, leading to reduced healthcare‐seeking behavior and service avoidance, ultimately compromising PHC effectiveness (Paulino et al. 2019; Costa‐Val et al. 2022; Oliveira et al. 2018).

Finally, proximal stressors refer to internal psychological and emotional processes that emerge as adaptive responses to adverse social environments. These include internalized LGBTQIA+ phobia, feelings of shame, anxiety, and fear associated with potential disclosure of sexual or gender identity, as well as identity concealment strategies aimed at protecting the individual from external threats (Ferreira and Bonan 2021; Silva et al. 2019). Additionally, gender dysphoria represents a key component of psychological distress experienced by some individuals, intensifying vulnerability (Ferreira and Bonan 2021; Ketzer et al. 2022). These internal stressors often lead to high‐risk behaviors, such as unsupervised hormone therapy and gender‐affirming procedures, exposing the population to additional health risks (Ketzer et al. 2022; Crenitte et al. 2023). Minority Stress Theory, considered in relation to Levesque's model of access to care, clarifies how individual experiences of stigma are deeply embedded within the wider dynamics of the health system. General stressors, such as structural inequalities and underfunded public services, intersect with Levesque's dimensions of accessibility, availability, and affordability by constraining the provision and visibility of inclusive primary health care services for LGBTQIA+ populations. Distal stressors—manifested through discriminatory encounters, cisheteronormative routines, and the absence of inclusive protocols—compromise the acceptability and adequacy of services, signaling to users that care is neither responsive nor safe. Proximal stressors, including internalized stigma, fear of disclosure, and identity concealment, directly affect individuals’ capacity and willingness to seek, reach, and engage with primary care, even when services are formally available. Taken together, these frameworks reveal how multilevel stress processes and dimensions of access reinforce one another, generating cumulative barriers while also highlighting strategic points for intervention, such as enhancing the acceptability and responsiveness of services to break cycles of exclusion.

The dynamic interaction among these three levels of stressors highlights the complexity of challenges faced by LGBTQIA+ individuals within Brazilian PHC, manifesting in negative health outcomes such as compromised mental health (including anxiety and depression), fragmented and discontinuous care, a narrow focus on sexually transmitted infections at the expense of other health needs, and increased vulnerability to chronic diseases and risks associated with informal care (Gomes et al. 2023; Mendes et al. 2023; Paiva et al. 2023). Furthermore, the lack of welcoming environments and professional unpreparedness exacerbate these deleterious effects, underscoring the need for strategies that promote inclusion, respect for diversity, and comprehensive care (Araújo et al. 2020; Costa‐Val et al. 2022; Torres et al. 2021; Oliveira et al. 2018).

### 4.5 Implications for Public Policy and Primary Health Care

This study yields significant implications for public policy and the structuring of PHC. First, the concentration of studies in the Southeast and Northeast regions of Brazil underscores the need for targeted investments in research capacity and monitoring systems in territories that have been historically underfunded. Strengthening graduate programs, research consortia, and dedicated public funding mechanisms for LGBTQIA+ health in these regions is essential to mitigate epistemic and territorial inequities in the production of evidence and in health services planning.

At the service level, our findings emphasize the need to transcend a narrow, HIV/AIDS‐centered approach to LGBTQIA+ health (Ketzer et al. 2022; Miskolci et al. 2022). National and state health authorities could incorporate explicit LGBTQIA+ health indicators, as well as fields for sexual orientation and gender identity, into PHC information systems, thereby enabling systematic monitoring of access, quality of care, and health outcomes across population groups. The development and implementation of inclusive clinical protocols and care pathways—encompassing mental health, sexual and reproductive health, gender‐affirming care, and chronic disease management—would support greater alignment between routine practice and the principles of the National Policy for Comprehensive LGBT Health (Costa‐Val et al. 2022).

Within the Family Health Strategy and other community‐based PHC models, policymakers may institutionalize training on sexual and gender diversity as a routine component of continuing professional development, rather than as isolated or one‐off initiatives (Vaz et al. 2024). This involves ensuring protected time, dedicated financial resources, and pedagogical support for team‐based training that addresses cisheteronormativity, intersectional stigma, and competencies in affirming communication.

Finally, the combined application of Minority Stress Theory and Levesque's model of access suggests that policy evaluation should extend beyond formal coverage or service availability to incorporate their effects on acceptability, continuity of care, and perceived safety among LGBTQIA+ users. Policy reforms that explicitly integrate these dimensions into planning, financing, and accountability frameworks are more likely to disrupt the cycles of stress, avoidance, and fragmented care documented in this study.

### 4.6 Implications for Nursing Practice and Nursing Policy

In primary health care (PHC) systems in Brazil and in many other countries, nurses are often the professionals who first interact with LGBTQIA+ users. The findings of our study suggest that nursing practice can play a transformative role in mitigating both distal and proximal stressors by creating care environments that are welcoming, respectful, and affirming. In everyday clinical encounters, nurses can actively challenge cisheteronormative assumptions, use inclusive language, avoid pathologizing expressions, and systematically explore health needs beyond HIV/STIs, including mental health, chronic conditions, reproductive health, and gender‐affirming care (Silva and Almeida 2024). Such practices directly contribute to increasing the acceptability and adequacy of PHC services for LGBTQIA+ populations (Silva and Almeida 2024).

From an educational perspective, undergraduate and graduate nursing curricula should integrate sexual and gender diversity as a transversal theme, articulated with ethics, human rights, and the social determinants of health (Yu et al. 2024). Simulation‐based training, case discussions, and community‐engaged learning experiences with LGBTQIA+ organizations may strengthen clinical judgment and cultural safety competencies (Matta et al. 2020).

In terms of policy and nursing leadership, professional councils, associations, and specialized bodies should advocate for regulatory standards that explicitly recognize the role of nursing in promoting LGBTQIA+ health equity. This includes incorporating LGBTQIA+ health indicators into quality assessment frameworks, supporting advanced nursing roles in community‐based and gender‐affirming models of care, and ensuring that nursing workforce planning reflects the needs of diverse populations (Yu et al. 2024). In the international sphere, the Brazilian experience—characterized by a robust public health system and community‐oriented PHC—offers lessons for other countries seeking to align nursing practice with equity‐driven PHC reforms.

Finally, nurses can act as key intermediaries between health services and LGBTQIA+ communities by engaging in participatory research, co‐designing interventions with service users, and strengthening social support networks. By leveraging their proximity to communities and their central role in multidisciplinary teams, nursing professionals are strategically positioned to translate the evidence generated by this scoping review into everyday practices that reduce minority stress, expand access, and improve health outcomes for LGBTQIA+ populations.

### 4.7 Limitations

This review has some limitations. By including only peer‐reviewed primary studies, we may have excluded grey literature and institutional or community reports that document innovative, community‐led initiatives in LGBTQIA+ health. Future reviews could benefit from incorporating broader sources to capture community‐based perspectives. The findings are restricted to what was reported in the included studies. While this may have limited the identification of other relevant experiences, it also underscores the need for more participatory and community‐led research with LGBTQIA+ populations. Regional representation was uneven, with the absence of studies from the Central‐West and minimal representation from the North. Although this restricts generalizability, it highlights priority areas for future research and policy action. Most studies were conducted with healthcare professionals, offering important insights into service provision, but resulting in the underrepresentation of LGBTQIA+ voices. Similarly, within LGBTQIA+ groups, more visible identities (lesbian, gay, and transgender) predominated, while bisexual, non‐binary, intersex, and other identities remained largely invisible. These imbalances reveal structural gaps in research agendas and point to the need for intersectional approaches. Finally, the predominant focus on conventional PHC settings limited the understanding of newer care arrangements (e.g., Family Health Strategy, Street Clinics). However, this gap itself is a relevant finding, as it draws attention to the need to explore diverse models of care. In sum, although methodological decisions imposed restrictions, they strengthened the rigor of the review and revealed important silences and inequities. These insights not only consolidate the current evidence but also guide future research toward more inclusive, intersectional, and context‐sensitive approaches.

## 5 Conclusion

The findings of this review highlight persistent structural, institutional, and epistemological barriers that restrict LGBTQIA+ populations' access to Primary Health Care (PHC) in Brazil. The complex interplay between general, distal, and proximal stressors contributes to negative health outcomes, such as worsening mental health, fragmented care, and increased vulnerability. Moreover, the lack of welcoming environments and unprepared health professionals exacerbates these harmful effects, perpetuating exclusion and suffering.

To ensure the health rights of this population, it is imperative to overcome normative biomedical models that historically invisibilize sexual and gender identity specificities. Expanding and diversifying scientific production to represent the varied experiences of LGBTQIA+ people, especially marginalized identities, is urgent to grant them voice and protagonism in research and knowledge construction. This expansion is crucial for grounding effective, contextualized, and sensitive interventions.

Full implementation of the National Policy for Integral Health of Lesbians, Gays, Bisexuals, Transvestites, and Transsexuals (PNSILGBT+) with adequate funding, inclusive protocols, and continuous evaluation is essential to promote equitable and quality access. Health professional training must integrate sexual and gender diversity transversally, encouraging ethical, intersectional, and welcoming practices recognizing individuals' unique trajectories.

Strengthening PHC as a strategic space for promoting equity and human rights, alongside fostering territorial and intersectional research that enhances representation and visibility of diverse identities, can drive transformative, evidence‐based actions. These actions reduce barriers, expand inclusive care, and improve mental and physical health outcomes for LGBTQIA+ populations. This integrated approach, grounded in Minority Stress Theory, guides policy and practice development to effectively address the multiple stressors and exclusions experienced by LGBTQIA+ people in the Brazilian PHC context.

## Policy on Using ChatGPT and Similar AI Tools

The authors used Gemini to review the quality of the English writing during the preparation of this work, without contributing to content creation. After using the tool, the authors carefully reviewed and edited the text as necessary and take full responsibility for the content of the publication.

## Funding

The authors have nothing to report.

## Conflicts of Interest

The authors declare no conflicts of interest.

## Public Involvement Statement

No public involvement in any aspect of this research.

## Guidelines and Standards Statement

This manuscript was drafted against the Preferred Reporting Items for Systematic reviews and Meta‐Analyses extension for Scoping Reviews (PRISMA‐ScR) checklist.

## Supporting information




**Appendix 1**: Search strategies applied in each database according to the PCC framework, with number of records retrieved (2018–2023).

## Data Availability

This study is a scoping review and no new data were generated. All analyses were based exclusively on secondary data from previously published and publicly available studies, which are fully cited in the reference list.
