# Supplementary material for: Access of the LGBTQIA+ Population to Brazilian Public Primary Health Care Services: A Scoping Review
Source: Public Health Nurs. 2026 Jan 12;43(2):497–510. doi: 10.1111/phn.70059 (PMC12968504; doi:10.1111/phn.70059)
Supplement: Supplementary file 1 — Appendix 1: Search strategies applied in each database according to the PCC framework, with number of records retrieved (2018–2023). [file PHN-43-497-s001.docx]

**Search strategies applied in each database according to the PCC framework, with number of records retrieved (2018–2023)**

| **Database/date** | **PCC** | **Search strategy** | **Number of articles** |
| --- | --- | --- | --- |
| MEDLINE/PubMed (via National Library of Medicine) (april, 2024) | **Participants** | ("Bisexuality"[MeSH Terms] OR "Homosexuality"[MeSH Terms] OR "Transsexualism"[MeSH Terms] OR "Sexual and Gender Minorities"[MeSH Terms] OR lesbian OR lesbians OR bisexual OR bisexuals OR transgender OR transgenders OR transsexual OR transsexuals OR queer OR queers OR intersex OR "men who have sex with men" OR MSM OR "sexual and gender minorities" OR nonheterosexual OR "non-heterosexual") | 167  records |
|  | **AND** | |  |
|  | **Concept** | ("Health Services Accessibility"[MeSH Terms] OR "Health Equity"[MeSH Terms] OR "Right to Health"[MeSH Terms] OR "Universal Health Care"[MeSH Terms] OR "Primary Health Care"[MeSH Terms] OR (health[Title/Abstract] AND (access[Title/Abstract] OR accessibility[Title/Abstract] OR equity[Title/Abstract] OR services[Title/Abstract] OR availability[Title/Abstract] OR inequality[Title/Abstract] OR utilization[Title/Abstract] OR usage[Title/Abstract]) |  |
|  | **AND** | |  |
|  | **Context** | ("Brazil"[MeSH Terms] OR Brazil OR "Minas Gerais" OR Bahia OR "Santa Catarina" OR Paraná OR "São Paulo" OR "Rio Grande do Sul" OR Ceará OR Goiás OR Maranhão OR Pernambuco OR Amazonas OR "Mato Grosso" OR "Rio Grande do Norte" OR Pará OR Piauí OR "Rio de Janeiro" OR "Espírito Santo" OR Paraíba OR "Mato Grosso do Sul" OR Acre OR Alagoas OR Roraima OR Sergipe OR Rondônia OR Tocantins OR Amapá OR "Distrito Federal") |  |
|  | **Limits** | Publication year: 2018-2023 |  |
|  | | | |
| EMBASE (Elsevier)  (april, 2024) | **Participants** | ('bisexuality'/exp OR 'homosexuality'/exp OR 'transsexualism'/exp OR 'sexual and gender minorities'/exp OR lesbian OR lesbians OR bisexual OR bisexuals OR transgender OR transgenders OR transsexual OR transsexuals OR queer OR queers OR intersex OR 'men who have sex with men' OR MSM OR 'sexual and gender minorities' OR nonheterosexual OR 'non-heterosexual') | 193  records |
|  | **AND** | |  |
|  | **Concept** | ('health care access'/exp OR 'primary health care'/exp OR 'health equity'/exp OR 'right to health'/exp OR 'universal health care'/exp OR (health NEAR/7 (access OR accessibility OR equity OR services OR availability OR inequality OR utilization OR usage))) |  |
|  | **AND** | |  |
|  | **Context** | ('brazil'/exp OR Brazil OR 'Minas Gerais' OR Bahia OR 'Santa Catarina' OR Paraná OR 'São Paulo' OR 'Rio Grande do Sul' OR Ceará OR Goiás OR Maranhão OR Pernambuco OR Amazonas OR 'Mato Grosso' OR 'Rio Grande do Norte' OR Pará OR Piauí OR 'Rio de Janeiro' OR 'Espírito Santo' OR Paraíba OR 'Mato Grosso do Sul' OR Acre OR Alagoas OR Roraima OR Sergipe OR Rondônia OR Tocantins OR Amapá OR 'Distrito Federal') |  |
|  | **Limits** | All fields; Source: Embase; 2018-2023 |  |
|  | | | |
| SCOPUS  (Elsevier)  (april, 2024) | **Participants** | (lgbt* OR homosexual* OR gay OR gays OR lesbian* OR bisexual* OR transgender* OR transsexual* OR queer* OR intersex* OR "men who have sex with men" OR msm OR "sexual and gender minorit*" OR non-heterosexual* OR nonheterosexual*) | 223  records |
|  | **AND** | |  |
|  | **Concept** | ("Health services accessibility" OR "access to primary care" OR "health equity" OR "right to health" OR "universal health care" OR (health W/7 (access* OR equit* OR service* OR availab* OR inequal* OR uses OR using OR usage))) |  |
|  | **AND** | |  |
|  | **Context** | (brazil* OR "minas gerais" OR bahia OR "santa catarina" OR parana OR "sao paulo" OR "rio grande do sul" OR ceara OR goias OR maranhao OR pernambuco OR amazonas OR "mato grosso" OR "rio grande do norte" OR para OR piaui OR "rio de janeiro" OR "espirito santo" OR paraiba OR "mato grosso do sul" OR acre OR alagoas OR roraima OR sergipe OR rondonia OR tocantins OR amapa OR "distrito federal") |  |
|  | **Limits** | TITLE-ABS-KEY; 2018-2023 |  |
|  | | | |
| CINAHL  (EBSCO)  (april, 2024) | **Participants** | (MH Bisexuality OR MH Homosexuality OR MH "Questioning Persons" OR MH "Transgender Persons" OR MH "Sexual and Gender Minorities") OR (LGB* OR homosexual* OR gay OR gays OR lesbian* OR bisexual* OR transgender* OR transsexual* OR queer* OR intersex* OR "men who have sex with men" OR MSM OR "sexual and gender minorit*" OR non-heterosexual* OR nonheterosexual*) | 162  articles (todos os campos, CINAHL with full text)  01/01/2018-31/12/2023) |
|  | **AND** | |  |
|  | **Concept** | (MH "Health Services Accessibility" OR MH "Right to Health" OR MH "Universal Health Care" OR MH "Primary Health Care" OR MH "Gender Equality") OR (health N10 (access* OR equit* OR service* OR availab* OR inequal* OR uses OR using OR usage)) |  |
|  | **AND** | |  |
|  | **Context** | (MH Brazil) OR (brazil* OR "minas gerais" OR bahia OR "santa catarina" OR parana OR "sao paulo" OR "rio grande do sul" OR ceara OR goias OR maranhao OR pernambuco OR amazonas OR "mato grosso" OR "rio grande do norte" OR para OR piaui OR "rio de janeiro" OR "espirito santo" OR paraiba OR "mato grosso do sul" OR acre OR alagoas OR roraima OR sergipe OR rondonia OR tocantins OR amapa OR "distrito federal") |  |
|  | **Limits** | All fields; source: cinahl with full text; 2018-2023 |  |
|  | | | |
| SciELO - Scientific Electronic Library Online (april, 2024) | **Participants** | (LGBT* OR homossexual* OR gay* OR lesb* OR bissexual* OR transgenero* OR transsexual* OR queer* OR intersex* OR "homens que fazem sexo com homens" OR HSH OR "minorias sexuais e de gênero" OR "minoria sexual e de gênero") | 211  records |
|  | **AND** | |  |
|  | **Concept** | ("Acesso aos Serviços de Saúde" OR "Acesso à Atenção Primária" OR "Equidade em Saúde" OR "Direito à Saúde" OR "Assistência de Saúde Universal" OR (saúde AND (acess* OR equidade OR serviço* OR disponibilidade OR desigualdade))) |  |
|  | **AND** | |  |
|  | **Context** | (Brasil* OR "Minas Gerais" OR Bahia OR "Santa Catarina" OR Paraná OR "São Paulo" OR "Rio Grande do Sul" OR Ceará OR Goiás OR Maranhão OR Pernambuco OR Amazonas OR "Mato Grosso" OR "Rio Grande do Norte" OR Pará OR Piauí OR "Rio de Janeiro" OR "Espírito Santo" OR Paraíba OR "Mato Grosso do Sul" OR Acre OR Alagoas OR Roraima OR Sergipe OR Rondônia OR Tocantins OR Amapá OR "Distrito Federal") |  |
|  | **Limits** | All indexes; 2018-2023 |  |
|  | | | |
| LILACS - Literatura Latino-americana e do Caribe em Ciências da Saúde (april, 2024) | **Participants** | ("Bissexualidade" OR "Homossexualidade" OR "Pessoas Transgênero" OR "Minorias Sexuais e de Gênero" OR "Homens que fazem sexo com homens" OR "Intersexo") | 284  records |
|  | **AND** | |  |
|  | **Concept** | ("Acesso aos Serviços de Saúde" OR "Acesso à Atenção Primária" OR "Equidade em Saúde" OR "Direito à Saúde" OR "Assistência de Saúde Universal") OR (saúde AND (acess* OR equidade OR serviço* OR disponibilidade OR desigualdade)) |  |
|  | **AND** | |  |
|  | **Context** | (Brasil* OR "Minas Gerais" OR Bahia OR "Santa Catarina" OR Paraná OR "São Paulo" OR "Rio Grande do Sul" OR Ceará OR Goiás OR Maranhão OR Pernambuco OR Amazonas OR "Mato Grosso" OR "Rio Grande do Norte" OR Pará OR Piauí OR "Rio de Janeiro" OR "Espírito Santo" OR Paraíba OR "Mato Grosso do Sul" OR Acre OR Alagoas OR Roraima OR Sergipe OR Rondônia OR Tocantins OR Amapá OR "Distrito Federal") |  |
|  | **Limits** | Title, abstract, subject; LILACS Plus Collection |  |
